# Insights into Binding Mechanisms of Potential Inhibitors Targeting PCSK9 Protein via Molecular Dynamics Simulation and Free Energy Calculation

**DOI:** 10.3390/molecules30142962

**Published:** 2025-07-14

**Authors:** Xingyu Wu, Xi Zhu, Min Fang, Fenghua Qi, Zhixiang Yin, John Z.H. Zhang, Shihua Luo, Tong Zhu, Ya Gao

**Affiliations:** 1 School of Mathematics, Physics and Statistics, Shanghai University of Engineering Science, Shanghai 201620, China; 18181045301@163.com (X.W.); zhuxi0032@163.com (X.Z.); 028123365@sues.edu.cn (M.F.); 21190006@sues.edu.cn (Z.Y.); 2 School of Electronic Engineering, Nanjing Xiaozhuang University, Nanjing 211171, China; qifenghua@njxzc.edu.cn; 3 Faculty of Synthetic Biology, Shenzhen University of Advanced Technology, Shenzhen 518107, China; john.zhang@nyu.edu; 4 Key Laboratory of Quantitative Synthetic Biology, Shenzhen Institute of Synthetic Biology, Shenzhen Institutes of Advanced Technology, Chinese Academy of Sciences, Shenzhen 518055, China; 5 NYU-ECNU Center for Computational Chemistry at NYU Shanghai, Shanghai 200062, China; 6 Department of Traumatology, Rui Jin Hospital, School of Medicine, Shanghai Jiao Tong University, Shanghai 200025, China; 7 Shanghai Engineering Research Center of Molecular Therapeutics & New Drug Development, School of Chemistry and Molecular Engineering, East China Normal University, Shanghai 200062, China

**Keywords:** binding free energy calculation, molecular dynamics simulation, virtual screening, small-molecule inhibitor, PCSK9 protein

## Abstract

The design of small-molecule inhibitors targeting proprotein convertase subtilisin/Kein type 9 (PCSK9) remains a forefront challenge in combating atherosclerosis. While various monoclonal antibodies have achieved clinical success, small-molecule inhibitors are hindered by the unique structural features of the PCSK9 binding interface. In this study, a potential small-molecule inhibitor was identified through virtual screening, followed by molecular dynamics (MD) simulations to explore the binding mechanisms between the inhibitor and the PCSK9 protein. Binding free energies were calculated using molecular mechanics/Generalized Born surface area (MM/GBSA) with the interaction entropy (IE) method, and critical hot-spot residues were identified via alanine scanning analysis. Key residues, including ARG237, ILE369, ARG194 and PHE379, were revealed to form critical interactions with inhibitor and play dominant roles during the inhibitor’s binding. In addition, the polarization effect was shown to significantly influence PCSK9–ligand binding. The identified inhibitor exhibited highly similar binding patterns with two known active compounds, providing valuable insights for the rational design and optimization of small-molecule inhibitors targeting PCSK9. This work contributes to the development of more effective treatments for hyperlipidemia and associated cardiovascular diseases.

## 1. Introduction

Atherosclerosis has emerged as one of the leading global health concerns in recent decades [1], contributing significantly to the burden of cardiovascular diseases, including coronary artery disease, stroke, and peripheral artery disease, which are the foremost causes of morbidity and mortality worldwide [2,3]. This chronic vascular disease is characterized by the accumulation of lipids, particularly cholesterol, along with other substances, on the arterial wall [4]. This process culminates in the formation of atherosclerotic plaques [5] that narrow and harden the arteries, thereby impeding blood flow and potentially precipitating severe cardiovascular events [6]. Elevated levels of low-density lipoprotein cholesterol (LDL-c) in the bloodstream are a primary driver of atherosclerosis [7]. LDL-c is normally eliminated from circulation via LDL receptors (LDLRs), which are predominantly located on hepatocytes [8]. These receptors internalize LDL particles for degradation in the liver and subsequently recycle the components to the plasma membrane. However, proprotein convertase subtilisin/Kein type 9 (PCSK9) [9], a protease primarily expressed in hepatocytes and secreted into bloodstream, serves as a negative regulator of LDLRs. By binding to LDLRs through protein–protein interactions (PPIs), PCSK9 facilitates the internalization of the LDLR–PCSK9 complex into hepatocytes, directing it to lysosomal degradation [10]. It reduces the availability of LDLRs on the cell surface, thereby elevating circulating LDL-c levels. Thus, inhibiting the PCSK9–LDLR interaction has emerged as a highly promising strategy for cholesterol-lowering therapies [11].

**Figure 1:**
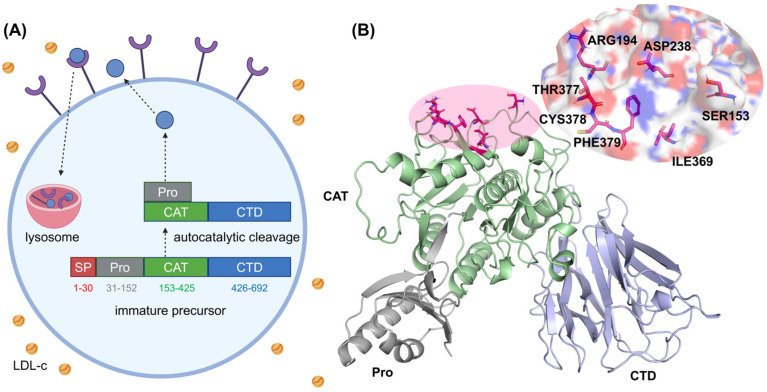
(**A**) An overview of PCSK9 synthesis and processing. Following its synthesis as a precursor in the endoplasmic reticulum, PCSK9 undergoes autocatalytic cleavage, exits the cell, binds to an LDLR, and directs the LDLR–PCSK9 complex for degradation in the lysosomes. (**B**) A structural representation of PCSK9, highlighting its prodomain, catalytic domain, and C-terminal domain. Key residues in the catalytic domain are emphasized for clarity.

PCSK9, the most recently identified member of the proprotein convertase family, undergoes complex post-translational modifications to achieve its functional form [12]. Its immature precursor contains an N-terminal signal peptide (SP, residues 1–30), a prodomain (Pro, residues 31–152), a catalytic domain (CAT, residues 153–451), and a C-terminal domain (CTD, residues 452–692) rich in cysteines [13], as shown in Figure 1A. PCSK9 undergoes autocatalytic cleavage between GLN152 and SER153 to form the mature fragment with the SP excised [14]. The structural basis for PCSK9–LDLR is well established based on detailed analysis of crystal structures. The PPI interface of PCSK9 shown in Figure 1B is characterized by a large, solvent-accessible, and relatively flat surface (~500 Å^2^) on the catalytic domain [15]. The interface comprises a network of hydrophobic residues, centered around PHE379, and several polar residues contributing to hydrogen bonding. Specifically, direct interactions between PCSK9 and LDLRs include a β-sheet-like contact unit (residues 377–379), hydrogen bonds involving SER153, ARG194, and ASP238, and hydrophobic interactions mediated by ILE369, CYS378, and PHE379. However, unlike traditional druggable targets such as enzymes, ion channels, and GPCRs, the absence of well-defined pockets or grooves on the PCSK9–LDLR PPI interface presents significant challenges for small-molecule drug design [16].

To date, several monoclonal antibodies (mAbs) targeting the PCSK9–LDLR PPI have demonstrated clinical success. Notably, Alirocumab and Evolocumab have received FDA approval for the treatment of heterozygous familial hypercholesterolemia (HeFH) [17]. Alirocumab, identified using hybridoma technology, binds to the catalytic domain of PCSK9 with a high affinity, effectively reducing LDL-c levels by up to 60% in patients with HeFH or at high cardiovascular risk [18]. Similarly, Evolocumab has been shown to achieve LDL-c reductions of 60–70% in clinical and preclinical studies [19]. Despite the success of mAbs, the widespread adoption of mAbs is constrained by factors such as injection-only administration, high production costs, limited shelf life, and potential immunogenic responses [20]. Consequently, small-molecule inhibitors are increasingly regarded as a more favorable modality for disrupting PCSK9–LDLR interactions. Recent advances in computational chemistry and structure-based drug design have accelerated the identification of small-molecule PCSK9 inhibitors [21,22,23]. Jaru et al. identified seven compounds that mimic key interface regions of LDLRs, demonstrating their binding affinity for PCSK9 and promising activity in cellular assays [24]. Sun et al. employed induced-fit docking [25] to generate a putative binding pocket on the PCSK9–LDLR PPI interface, enabling the virtual screening of a 250,000-compound library and the selection of a promising lead compound [26]. Similarly, Lammi and colleagues designed a tetraimidazole derivative, RIm13 (hereafter referred as RIm) using structure-based approaches, which exhibited inhibitory activity against PCSK9–LDLR binding, albeit with limited selectivity and stability [27]. SBC-115076 (hereafter referred as SBC), discovered through PPI surface modeling and high-throughput virtual screening, effectively reduced PCSK9 activity and upregulated LDLR expression in hepatocytes [28]. Although several small peptides, peptidomimetics, and small molecules targeting PCSK9–LDLR PPIs have been reported, a deeper understanding of the interaction mechanisms between PCSK9 and small molecules is necessary to guide future drug development.

In this study, we employed a comprehensive computational approach that integrates virtual screening [29], molecular dynamics (MD) simulations [30], and binding free energy calculations [31] to identify novel small-molecule inhibitors targeting PCSK9. In addition, two known active inhibitors, SBC-115076 and RIm13, are included for comparative analysis. Our results identify a promising small-molecule candidate with strong binding affinity for PCSK9, exhibiting similar binding patterns with the two known active inhibitors. This work provides valuable insights into the design of small-molecule inhibitors targeting PCSK9, establishing a foundation for subsequent experimental validation and optimization of these compounds for the treatment of hyperlipidemia and related cardiovascular diseases.

## 2. Results and Discussion

### 2.1. Virtual Screening

**Figure 2:**
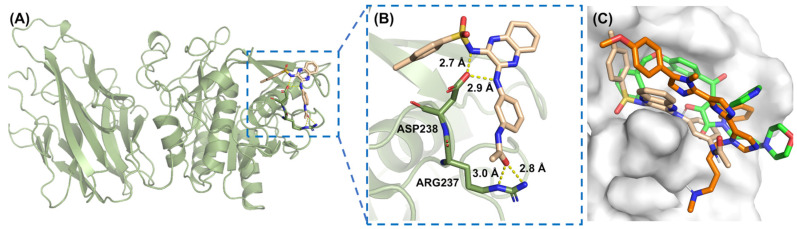
(**A**) Predicted binding mode of the PCSK9 protein and ligand AK. The ligand is colored with light wheat. (**B**) A close-up view of the binding interface. (**C**) The conformational alignment of three molecules in binding pocket, SBC (green), RIm (orange), AK (wheat). All structures are plotted using PyMOL 2.5.7 software.

After high-throughput virtual screening (HTVS), 14,236 molecules were initially selected, which were then refined to 1423 molecules through standard-precision (SP) screening. Finally, extra-precision (XP) screening further narrowed the pool to 142 candidates. During the three stages, certain molecules with an unreasonable geometric structure or chemical constraint were excluded. In AutoDock Vina, the top 5000 molecules were selected based on their docking scores. A comparative analysis was conducted to ensure consistent results in both methods by matching the molecular IDs. Among the 142 molecules from XP screening, the first common candidate, AK-968/12164422 (hereafter referred as AK) was identified. For this candidate, the docking score with PCSK9 under XP precision was −5.9 kcal/mol, and in AutoDock Vina it was −8.0 kcal/mol, indicating comparatively strong binding affinity across two different docking precisions. To better assess the binding affinity of the candidate, the docking scores of two known active inhibitors (SBC and RIm) were also obtained using the same XP precision scoring function, with −4.3 kcal/mol for RIm and −4.6 kcal/mole for SBC, which are a little lower than the identified inhibitor AK. The binding pose of this candidate with PCSK9 protein is illustrated in Figure 2A, and the key residues ARG237 and ASP238 that interacted with AK in the binding pocket are shown in Figure 2B. A network of hydrogen bonds between the protein and the AK molecule, specifically involving ASP238 OD2 with AK N2 (2.9 Å) and AK N4 (2.7 Å), as well as ARG237 NE with AK O3 (3.0 Å) and ARG237 NH2 with AK O3 (2.8 Å), highlights significant electrostatic interactions that strengthen the protein–ligand association. Figure 2C shows the alignment of the three ligands (SBC, RIm, and AK) within the binding pocket, and similar binding poses were detected between the ligands and the protein, indicating the reliability of the small-molecule AK selected by virtual screening compared to the two known active inhibitors.

### 2.2. ADMET Prediction

The absorption, distribution, metabolism, and excretion (ADME) are important properties of the selected hits. SwissADME [32], a free web tool for pharmacokinetic profiling, provides critical parameters including lipophilicity, water solubility (Log S), drug-likeness rules, and medicinal chemistry friendliness based on input SMILES data. The predicted results of three inhibitors are shown in Table 1. The results show that all three inhibitors were moderately soluble molecules as the Log S values were in the range of −6 to −4. The higher molecular weights of SBC and RIm (527.61 and 495.62 g/mol, respectively) and the smaller molecular weight of AK (447.52 g/mol) may give AK an advantage in permeation through cellular membranes, especially in oral absorption. For the topological polar surface area (TPSA), which is a key predictor of passive membrane permeability and solubility, AK displays the highest TPSA (121.46 Å^2^), followed by SBC (92.2 Å^2^) and RIm (74.72 Å^2^). The elevated TPSA for AK molecule, typically associated with reduced passive diffusion, might offset its MW advantage. AK has a higher number of hydrogen bond acceptors (eight) and hydrogen bond donors (three), which may allow it to form more hydrogen bonding interactions when binding to targets. The Log P value of AK is 3.31, which is similar to other two molecules, indicating its moderate lipophilicity. SwissADME also predicts skin permeability of the molecules. The predicted values of three compounds are highly negative (log Kp), which means that these compounds are not permeable through skin. Overall, the ADME properties of AK are similar to those of SBC and RIm and have the potential to be further optimized and investigated in drug development targeting the PCSK9 protein.

The prediction of compound toxicities within silicon is an important part of the drug design. To determine the toxicity and adverse effects of the three inhibitors, ADMETsar (https://lmmd.ecust.edu.cn/admetsar1/predict, accessed on 29 April 2025) was used to predict the toxicities of the three small molecules. As shown in Table 1, it revealed that all three molecules are non–AMES toxic, and the predicted acute oral toxicity showed that the compounds belonged to the class III category, which includes compounds with an LD_50_ in the range of 500–5000 mg/kg, meaning that these compounds do not produce acute oral toxicity in the 500 mg/kg concentration range. Moreover, none of the compounds are carcinogenic, indicating that these compounds possess good drug-likeness. The predicted rat acute toxicity (LD_50_ in mol/kg) was also similarly low for SBC (2.70), RIm (2.76), and AK (2.23), suggesting that these compounds are not lethal up to concentrations > 2 mol/kg. The pharmacokinetic and physiological properties of the AK molecule indicates that it is suitable for molecule design targeting PCSK9 protein.

### 2.3. Structural Stability

**Figure 3:**
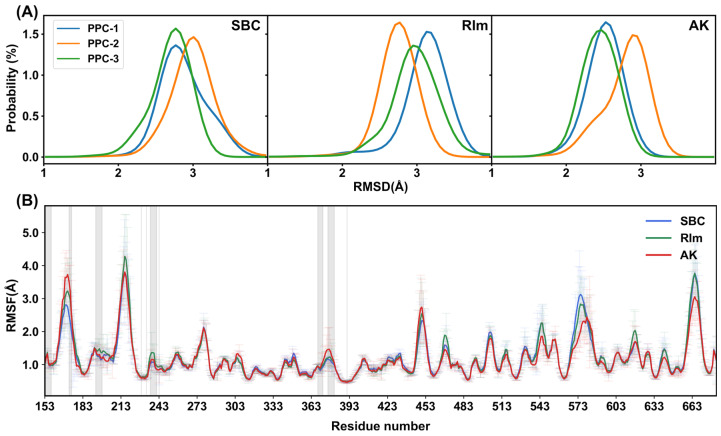
(**A**) Backbone RMSD probabilities for the PCSK9–SBC, PCSK9–RIm, and PCSK9–AK systems under the PPC model. Replicas 1, 2, and 3 are colored with blue, orange, and green, respectively. (**B**) RMSFs including standard errors of Cα atoms in each residue in the three PCSK9–inhibitor complexes. The residues within 5 Å of the active site are shaded.

In order to quantitatively characterize the conformational stability, the root mean-square deviation (RMSD) of all heavy atoms (C, N, and O) in the protein and ligand were calculated for each system. However, during simulations using the general Amber force field, ligands were observed to escape occasionally from the binding pocket, indicating the unstable interaction between the protein and ligand, which was also detected in Sun’s work [26]. In our previous studies, the polarization effect has proved to be a critical factor affecting the stability of the binding between the protein and ligand [33,34,35]. To address the fundamental limitation of fixed-charge force fields, we implemented the PPC model [23,36,37], which is obtained by quantum mechanical calculation of the entire protein in solvent using a fragment approach combined with a continuum solvent model and explicitly accounts for ligand-induced charge redistribution in the protein binding site. Thus, the PPC contains the proper electrostatic polarization effect and provides a more reliable description for the protein structure and dynamics, which is particularly critical for polar residues like ASP238 and ARG237 that mediate key interactions with PCSK9 inhibitors. In this work, the PPC model was used in simulations to include the polarization effect caused by the binding of the ligand. During 200-ns simulations for each system under the PPC model, the complex gradually reached stabilization in each replica shown in Figure S1. The RMSD values of the protein in respect to the initial structure fluctuated in the range of 1.5–4.0 Å, indicating a stable complex structure binding with the ligand. The RMSD distributions in the three replicas for each complex are shown in Figure 3A, and the average RMSDs for SBC, RIm, and AK are 2.86, 2.94, and 2.58 Å, respectively, indicating compact and stable complex conformation formed during simulations. The escape of ligands from the binding pocket was not observed, which is consistent with the fact that the incorporation of the polarization effect could stabilize the protein–ligand complex interaction. Root mean-square fluctuations (RMSFs) of the PCSK9 residues were also calculated to measure the protein’s fluctuation in the PPC model during the MD simulations, as shown in Figure 3B. Most residues exhibited a low fluctuation, indicating the structural stability of the complexes during simulations. RMSFs of the residues within 5 Å of the active site are highlighted with the shaded regions to provide a more detailed view near the active site, including residues 153–158, 170–175, 193–198, 236–241, 368–372, and 376–381. We found that RMSF values of these regions dominantly remain below 2.0 Å. Only RMSF values of the region 170–175 fluctuated between 2.0 and 4.0 Å, which corresponds to the loop structure constructed with the Prime module. This loop region did not form hydrogen bonding or hydrophobic interaction with small molecules, resulting in a flexible and dynamic structure. The most pronounced fluctuations occurred in regions such as residues 213–218, 570–580, and 660–680, which are loops far from the active site. The higher fluctuations in these regions are caused by their more dynamic structural characteristics. In addition, the PCSK9–AK complex shows comparatively similar fluctuations compared to the known active inhibitors PCSK9–SBC or PCSK9–RIm, indicating the ability of the small molecule AK for stabilizing the PCSK9 protein.

### 2.4. Interaction Patterns

**Figure 4:**
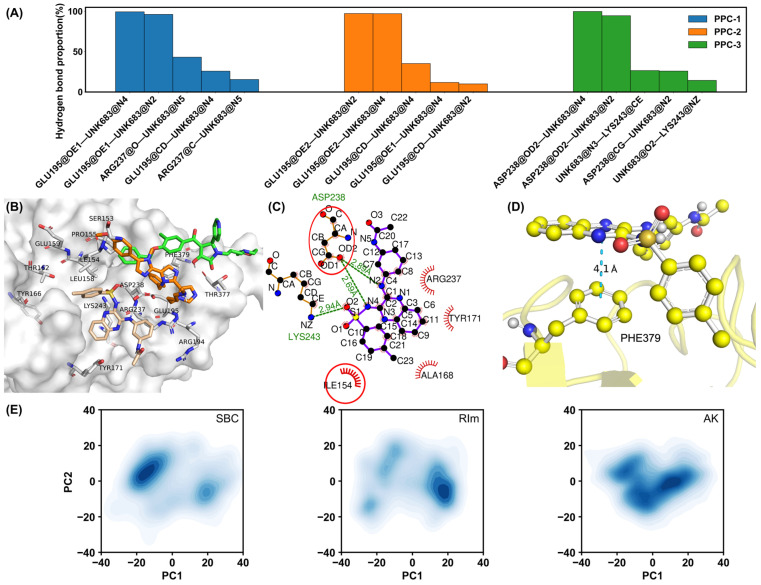
(**A**) Hydrogen bond proportion in each replica for the PCSK9–AK system in the PPC model. (**B**) Superposition of representative structures from the most populated conformational clusters for the SBC (green), RIm (orange), and AK (wheat) systems. (**C**) Critical interactions identified by LigPlot [39] between PCSK9 and the small molecule AK in the representative structure. Green dashed lines represent hydrogen bonds with distances. The red “eyelash” symbols denote hydrophobic interactions, specifically between the hydrophobic amino acid side chains and small molecules. The residues in the red circle are also observed to form interactions in other two molecules. (**D**) π–π stacking interaction formed between the PHE379 residue and the small molecule AK. (**E**) Two-dimensional projection map to two eigenvectors of all three systems.

To understand the interaction mechanisms between the PCSK9 protein and each ligand, hydrogen bond and interaction patterns were analyzed. Figure 4A presents the hydrogen bond occupancy in the three replicas for the PCSK9–AK system. Hydrogen bonds formed with residues GLU195 and ASP238 exhibit higher frequencies in all three replicas, suggesting the two residues play a critical role in stabilizing the protein–ligand interaction. The averaged proportion of the top three hydrogen bonds reached 98.90%. For the SBC molecule, hydrogen bonds with GLU195, THR377, ARG237, and PHE379 also formed, as shown in Figure S2, with a significant proportion. However, only up to a 40% proportion of the hydrogen bonds between the protein (SER381) and the RIm molecule formed during the PPC simulations, as shown in Figure S3, indicating the weak electrostatic interaction between the protein and RIm molecule. It is also indicated by the weak ΔE_ele_ in the binding free energy calculations. Therefore, other critical interactions between the protein and small molecules should maintain the integrity of the complex structure. To characterize the key interactions governing the binding of small molecules, clustering analysis on 75,000 frames from the three replicas was performed for each complex system to identify the most populated binding conformations, which is based on backbone RMSDs using a K-means algorithm, providing a more meaningful characterization of the binding modes. The population of the most populated cluster is 34.3% (PCSK9–SBC), 33.5% (PCSK9–RIm), and 33.3% (PCSK9–AK. A representative structure (the frame closest to the geometric center of the cluster) for each system was aligned and shown in Figure 4B for comparison, in which the three small molecules exhibit distinct spatial distributions within the binding pocket after 200-ns simulations, suggesting different binding affinities of the three molecules and the divergent interacting networks underlying the binding. Notably, the population of the second populated cluster is comparable to that of the most populated cluster across all systems, but different binding modes were observed compared to that in the most populated cluster, shown in Figure S4. Figure 4C illustrates the interactions, including hydrogen bonding and hydrophobic contacts for the representative structure of the PCSK9–AK complex from the most populated cluster. The red circles indicate the hydrophobic or π–π stacking interactions, in which the ILE154, ARG237, LYS243 and ASP238 residues play critical roles. For the representative structures binding with small molecules SBC and RIm, similar residues were observed forming hydrophobic interactions with the ligands, shown in Figures S2B and S3B, which is consistent with the key residues around the binding pocket. Furthermore, to clarify whether the binding modes observed during the MD simulations are consistent with the initial docking poses, the superposition of the initial docking pose and two representative structures from the most two populated clusters is shown in Figure S5 for each complex system. For all three systems, the binding poses from one of populated clusters remain consistent with their initial docking poses, confirming the reliability of the predicted binding modes considering the comparable population of the most frequent two clusters. Notably, the PCSK9–RIm complex structure is primarily stabilized by hydrophobic and π–π stacking interactions, as indicated by the significantly lower probability of hydrogen bonding observed between PCSK9 and the RIm molecule during simulations. It is also proved by following free energy calculations, in which the van der Waals energy term contributes significantly to the binding energy in PCSK9–RIm system. All three compounds were found to form a similar π–π stacking interaction with residue PHE379. It is illustrated in Figure 4D, in which the π–π stacking interaction between residue PHE379 and the small molecule AK is shown as a representative example. The interaction pattern analysis indicates that polar and nonpolar interactions are both critical factors maintaining the stability of the protein–ligand complex structure.

Principal component analysis (PCA) [38] was also performed to characterize the dominant protein motions across all three systems, which was carried out using the cpptraj module in AmberTools, with trajectories aligned to the initial Cα atom coordinates by RMS fitting. The covariance matrices were constructed from multiple independent replicas (25,000 frames per replica, in total 75,000 frames) and diagonalized to extract eigenvectors and eigenvalues, representing the principal components (PCs) of motion. Specifically, for the PCSK9–AK complex, the first three principal components (PC1, PC2, and PC3) accounted for 34.0%, 15.9%, and 11.7% of the total conformational variance, respectively. In comparison, the SBC system exhibited 36.9%, 18.8%, and 13.6%, while the RIm system showed 36.0%, 19.4%, and 11.5%. The results indicate that the dominant protein motions in all three systems are effectively captured within the first three principal components, underscoring their significance in describing the essential conformational dynamics. Then, 75,000 frames for each complex system were projected onto the first two eigenvectors, which was used to further explore the conformational differences of the PCSK9 protein under different inhibitors binding. As shown in Figure 4E, three complex systems exhibited distinct conformational diversity, with their principal components distributed differently in the PC1/PC2 space. When binding with the SBC or RIm molecule, the PCSK9 protein adopted different conformational clusters, indicating that the two molecules induced discrepancies in the movement of the PCSK9 protein. When the small molecule AK binds to the PCSK9 protein, the conformation is mainly concentrated in one conformational cluster, showing better stability. The different binding affinity of the three small molecules toward the PCSK9 protein may be caused by their different structural features. RIm possesses a unique structure characterized by a fused bis-imidazole core and multiple aromatic rings, as well as a long aliphatic side chain. Compared to SBC and AK, RIm contains the highest number of aromatic rings, which can facilitate extensive π–π stacking interactions with aromatic residues in the PCSK9 binding pocket. In addition, the long alkyl side chain and benzyl group provide significant hydrophobic surface area, enabling strong van der Waals and hydrophobic interactions with nonpolar regions of the protein. This structural feature may explain why RIm retains biological activity despite its low hydrogen bond occupancy.

**Figure 5:**
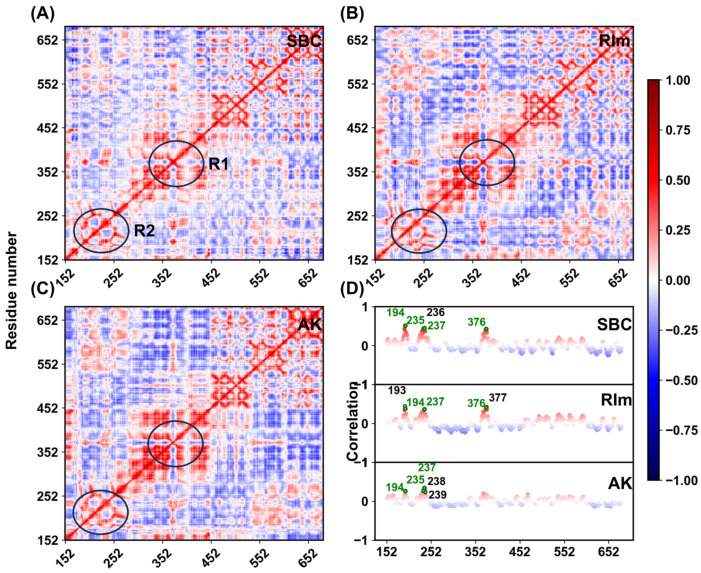
The DCCMs were calculated by the coordinates of the Cα atoms for the protein binding with (**A**) SBC, (**B**) RIm, and (**C**) AK. (**D**) Correlation of the PCSK9 residues in the three systems. The top five residues with the highest correlation are labeled.

To further elucidate the internal dynamics of the three inhibitors on the movement of the PCSK9 protein, the DCCMs of the protein residues throughout the simulations were calculated. As shown in Figure 5, the positive and negative correlations are marked in red (1.0) and blue (−1.0), respectively. The diagonal regions indicate the movement of each residue relative to itself. Overall, the correlations of certain residues in the three systems are similar with each other, suggesting that these inhibitors have similar effects on the corresponding regions of the protein. Residues near the binding pocket are highlighted with R1 and R2 circles, showing highly correlated motions in all three complexes. Near the R1 region, the movement correlation of the protein residues was significantly higher in the AK system than in other two systems, suggesting that the protein residues are subjected to stronger positive correlations with the ligand in this region. Meanwhile, both the red and blue colors of this system are darker than other two systems, implying that the binding of the small molecule AK to PCSK9 stimulates stronger correlated or anticorrelated motions. To further analyze the interaction of the protein residues with the inhibitors, a list of correlations between the protein and inhibitor molecules in the three systems were extracted. The top five residues with the highest correlation of protein residues with the inhibitors in each system were labeled, as shown in Figure 5D. In the three systems, common residues ARG194, SER235, ARG237, and SER376 show strong positive correlations, which were also the residues forming hydrogen bonds and hydrophobic interactions with the protein at the binding interface. In conclusion, the binding of different ligands has a significant impact on the dynamic behavior of the protein, especially on the residues near the binding pocket. In the other two replicas shown in Figure S6, protein residues with the highest correlation were ARG194, ARG237, LEU376, and THR377, showing a consistency across the three replicas and identifying the critical role of these residues in the binding process.

### 2.5. Binding Free Energy Calculations

To predict the strength of the inhibitor’s tendency to interact with PCSK9, the binding free energy of PCSK9 with each inhibitor was calculated. The calculated binding free energies for the three complexes are listed in Table 2. Here, we also included simulations using the general AMBER force field for comparison. Under AMBER force field simulations, the averaged enthalpy contribution to the binding free energy is −19.90, 1.24, and −23.03 kcal/mol for the SBC, RIm, and AK molecules, respectively, in which the electrostatic and van der Waals terms make significant contributions. It is noteworthy that an unfavorable enthalpy contribution is detected in PCSK9–RIm complex when compared to the SBC and AK inhibitors, with a high positive polar solvation energy $\Delta G_{gb}$. The calculated binding free energies are −0.56, 48.47, and 7.31 kcal/mol when the entropic contribution is included, which was calculated using the interaction entropy method. The entropic contributions in the three systems are 19.34, 47.23, and 30.34 kcal/mol, respectively, indicating a severe interaction energy fluctuation and unfavorable binding poses during the non-polarizable AMBER force field. The binding free energies calculated using trajectories generated from AMBER force field indicate that the protein could not describe the protein–ligand interaction correctly, especially for the large and relatively flat PPI surface, resulting in unfavorable binding free energies.

**Figure 6:**
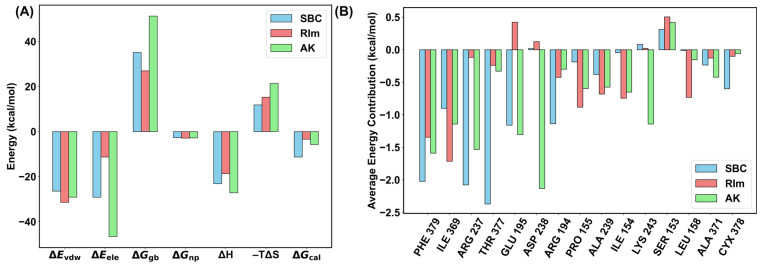
(**A**) The binding free energy and each energy term of three simulated complexes in the PPC model. (**B**) Decomposition of the binding free energy for each residue in three systems using the MM/GBSA method.

When the polarization effect was included, the enthalpy contributions are −23.15, −18.67, and −27.18 kcal/mol, decreasing by 3.25, 19.91, and 4.15 kcal/mol compared to that in the AMBER force field. The improved binding affinity originates from the stronger electrostatic interaction, except for the small-molecule RIm, for which it has been indicated that hydrophobic interactions mainly maintain the stability of the PCSK9–RIm structure. However, the polar solvation energy, which describes the polar components of the desolvation free energy, decreased by 24.27 kcal/mol in PCSK9–RIm system. The entropic contributions in three systems are 11.86, 15.28, and 21.41 kcal/mol, respectively, which are decreased by 7.48, 31.95, and 8.93 kcal/mol compared with that in AMBER force field, indicating more stable interaction modes sampled when incorporating the polarization effect. The averaged binding free energy and each energy term in PPC model were also shown in Figure 6A, in which both electrostatic and van der Waals interactions are key positive contributors to the binding free energy compared to the polar solvation energy and entropic energy. Among all three systems, the binding affinity between PCSK9 and the small molecule SBC is the strongest, with the energy of −11.28 kcal/mol; the binding affinities of the RIm and AK systems are close to each other with −3.39 and −5.77 kcal/mol, respectively. This suggests that due to the unique structural features of the PCSK9 binding interface, the binding affinity towards PCSK9 is not especially strong across these inhibitors, proposing the challenge of designing small molecule targeting PCSK9. From the binding free energy calculations, the polarization effect is critically necessary for describing the binding pose and affinity, especially for the large and relatively flat PPI surface, and the binding affinity is strengthened along with the favorable electrostatic interaction formed when the polarization effect is included.

To gain a deeper understanding for the source of the difference in the binding affinity among the three inhibitors, the contribution of each residue to the binding free energy was calculated. In this work, residues with a binding free energy contribution exceeding −1 kcal/mol were identified as hot-spot residues. The results are shown in Figure 6B, in which the energy contribution of the residues near the binding pocket were calculated. For the PCSK9 protein bound to the SBC molecule, the PHE379, ARG237, THR377, GLU195, and ARG194 residues, five in total, were identified as hot-spot residues. For the PCSK9 protein binding with the RIm molecule, only two residues, PHE379 and ILE369, were identified as hot-spot residues. For the AK molecule, the energy contributions of the PHE379, ILE369, ARG237, GLU195, ASP238, and LYS243 residues were greater than −1 kcal/mol. The number of hot-spot residues in the PCSK9–RIm system was less than other two systems, resulting in a weak binding affinity for the RIm molecule compared to the SBC and AK molecules, in which more complex interactions formed and lead to more residues in the PCSK9 protein participating in the binding process. Residue PHE379 was detected as a critical residue among the three systems. It is consistent with the above analysis, in which all three molecules formed a π–π stacking interaction with PHE379. Residues ARG237 and GLU195 are the common hot-spot residues for the protein bound to the SBC and AK molecules, which also agrees that the two residues exhibiting a high frequency of hydrogen bonding in all three replicas.

To evaluate the energy contribution of each residue in detail, especially residues in the binding interface region, an ASIE approach was utilized to analyze the energetic contributions of individual residues within 5 Å of the binding surface, in which each residue is mutated to alanine and then the contribution of a specific residue to binding energy is assessed ($\Delta \Delta G_{bind}^{x\to a}$). The calculated results for each residue are shown in Table S1. For the hot-spot residues in PCSK9–SBC system, the averaged ΔΔG_bind_ values over the three replicas of each residue are −1.70 (PHE379), −2.58 (ARG237), −1.62 (THR377), −1.22 (GLU195), and −1.07 (ARG194) kcal/mol, while for PCSK9 binding with RIm molecule, only residue ARG194 was classified as a hot-spot residue, with a ΔΔG_bind_ of −1.25 kcal/mol. For the PCSK9–AK system, the hot-spot residues included GLU195, ARG237, ASP238, and LYS243 with ΔΔG_bind_ values of −7.66, −1.00, −1.55, and −1.88 kcal/mol, respectively. The identified hot-spot residues are consistent with the results of the energy contribution of the residues, highlighting their potential as key residues for optimizing binding affinity in inhibitor design. Considering alanine is a simple amino acid with a methyl side chain and its contribution to the free energy is negligible, the energy contribution of each residue within 5 Å of the binding surface to the energy difference before and after mutation ${\Delta \Delta G}_{bind}^{x\to a}$ was evaluated. The total energy contribution of these residues to the binding affinity in PCSK9–RIm system was −4.16 kcal/mol, which is significantly distinctly lower than the energy contribution of the residues in other two systems (PCSK9–SBC: −9.15 kcal/mol, PCSK9–AK: −13.47 kcal/mol). It revealed that the hot-spot residues in the protein, which either directly or indirectly participate in the binding interface, significantly impact and determine the binding affinity between the protein and inhibitor.

## 3. Methods

### 3.1. Structures Preparation and Virtual Screening

**Figure 7:**
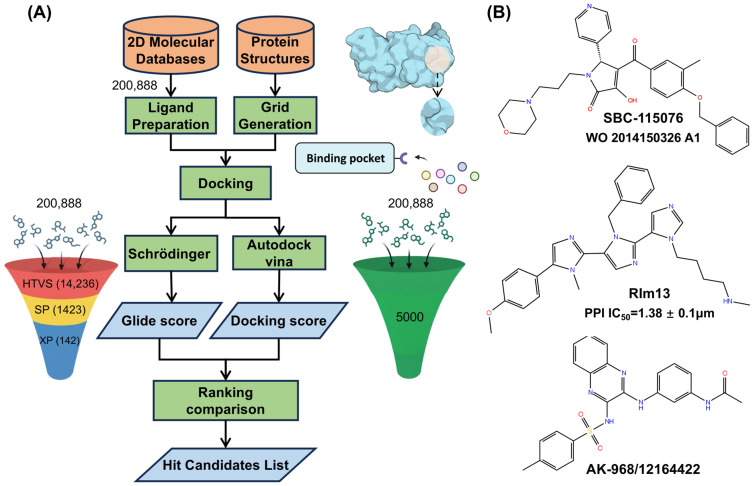
(**A**) Chemical structures of SBC-115076, RIm13, and hit candidate molecule AK-968/12164422 identified by virtual screening methods. (**B**) The procedure in the virtual screening for identifying promising drug candidates.

Two different tools, Schrödinger suite (release 2021–3) [40] and AutoDock Vina (24 December 2017) [41], were used for virtual screening based on molecular docking to enhance the reliability of the results. Figure 7A shows the procedure of virtual screening for identifying the promising small-molecule candidates. The Specs compound library (https://www.specs.net), containing a diverse set of 200,888 small molecules, was used as the 2D molecule databases. The crystal structure of the PCSK9 protein (PDB ID: 2P4E) was obtained from the protein data bank [42]. The protein structure was refined using the protein preparation wizard module in Schrödinger [26,43], including adding hydrogen atoms, assigning partial charges, and determining the protonation states. Following removal of the signal peptide (residues 1–30) and the prodomain (residues 31–152), six discontinuous segments in Chain A (residues 169–175, 213–218, 450–452, 573–584, 660–667, and 683–692) were identified as missing in the electron density map and were constructed using the Prime module [44] through knowledge-based loop modeling with fragment libraries.

The PPI interface [45] was defined well in the crystal structure of the PCSK9–LDLR EGF-A complex (PDB: 3BPS), and the binding pocket was generated with Schrödinger’s Induced-fit docking (IFD) module using the known active small molecule SBC (shown in Figure 7B) based on the PPI interface of the PCSK9–LDLR complex. The grid center was set to the centroid of PCSK9 residues ARG194, ASP238, and SER153, which form interaction contacts with the LDLR EGF-A domain [45]. A cubic grid box of 20 Å in edge length was used to enclose the entire binding site. In addition, another known active molecule, RIm13 (shown in Figure 7B), was also docked to PCSK9 to investigate its binding mechanism comparatively. The two known active molecules were initially modeled and optimized using Gaussian16 at the M06-2X/6-311G (2d, p) level [46]. The obtained structure of PCSK9 bound to SBC was used for virtual screening against the prepared Specs compound library, with the binding pocket around key residues SER153, ARG194, ASP238, ILE369, CYS378, and PHE379 shown in Figure 1B. The virtual screening workflow in Schrödinger involves several steps: ligand preparation using LigPrep, filtering with QikProp as well as Lipinski’s rules, high-throughput virtual screening (HTVS), and standard-precision (SP) and extra-precision (XP) docking [47]. At each stage, only the top 10% of the generated poses are retained, as shown in Figure 7A, ensuring the most promising candidates are selected. AutoDock Vina follows a similar fundamental process as Schrödinger [48]. The exhaustiveness parameter was set to 4. Finally, the hit candidate AK-968/12164422, shown in Figure 7B, exhibited a good binding affinity across both independent virtual screening platforms and was identified for further study.

### 3.2. Molecular Dynamics Simulation

The complex structure was immersed in a cubic box using the TIP3P water model, ensuring a minimum solute–wall distance of 12 Å. Counterions were added to neutralize the system. The AMBER ff14SB [49] force field was applied to model the protein, while ligand force field parameters were generated using the general AMBER force field (GAFF) [50], with atomic charges assigned via the AM1-BCC method [51]. The protonation states of all titratable residues were assigned using the Protein Preparation Wizard module [43] in Schrödinger, with the target pH set to 7.0 ± 2.0. To eliminate unfavorable contacts prior to simulation, we conducted energy minimization using 20,000 steps of the steepest descent followed by 40,000 steps of conjugate gradient methods. Throughout the minimization and equilibration phases, positional restraints with a force constant of 2 kcal/mol·Å^2^ were applied to the protein backbone for maintaining the structural stability. During the production run, no positional restraints were applied. The relaxed structure was gradually heated to 300 K over 50 ps, with all protein atoms restrained by a force constant of 2 kcal/mol·Å^2^. The SHAKE algorithm [52] was employed to fix all bonds involving hydrogen atoms. Electrostatic interactions were calculated using the particle mesh Ewald method with a 10 Å cutoff in real space. Temperature was regulated at 300 K using a Langevin thermostat [53] with a collision frequency [54] of 1.0 ps^−1^, while isotropic pressure coupling with a relaxation time of 2 ps maintained the pressure to 1 atm. The integration time step was set to 2 fs. To enhance sampling and assess simulation convergence, three independent replicas with different random seed numbers were generated for each system. Trajectories were recorded every 10 ps, and MD simulations were extended to 200 ns for each replica. For simulations employing polarized protein-specific charges (PPC), the derived PPCs were used by substituting the conventional AMBER force field charges for solute. All simulations were performed using the PMEMD program in Amber22, with 25,000 frame snapshots from the last 100 ns of each replica used for subsequent analysis.

### 3.3. Polarized Protein-Specific Charge (PPC)

The PPC [55] method combines molecular fragmentation with conjugate caps (MFCC [56]) strategy and the Poisson–Boltzmann (PB [57]) solvation model to enhance the accuracy of the quantum mechanical (QM) calculations for protein–ligand systems in solution. First, the MFCC technique partitions the protein into discrete amino acid fragments, enabling the QM computations to determine their electronic density distribution with precision. The RESP [58] program then refines the atomic charges by fitting them to the electrostatic potential derived from these fragments. Solvation interactions, including solvation energy and charge redistribution at the complex–solvent boundary, are analyzed by solving the PB equation. This integrated approach ensures that residue and surface charges collectively account for solvent effects, creating an appropriate environmental for the subsequent QM calculations. By iterating this process, polarization interactions between the solute and solvent stabilize the solvation energy of the protein, offering a comprehensive depiction of its solvation characteristics. The derived polarized protein-specific charges are then introduced into MD simulations by replacing the solute charges with the PPC.

### 3.4. Dynamic Cross-Correlation Matrices (DCCM)

The DCCM [59] between residues i and j, $C_{ij}$, is given by $C_{ij}=\frac{<∆r_{i}\cdot {∆r}_{j}>}{\sqrt{<∆r_{i}^{2}><∆r_{j}^{2}>}}$, where ${∆r}_{i}$ is the instantaneous displacement of residue i from its average position. The cross-correlation values range from +1 (perfectly correlated motion) to −1 (perfectly anti-correlated motion), where positive values indicate that the residues move in the same direction, and negative values indicate movement in opposite directions. The DCCM analysis was performed using the cpptraj program in the Amber22 package.

### 3.5. The Binding Free Energy Calculation

The binding free energy ($\Delta G_{bind})$ between PCSK9 and the ligand was determined by integrating the Molecular Mechanics Generalized Born Surface Area (MM/GBSA) [60] approach for enthalpic contributions and the interaction Entropy (IE) [61] method for the entropic effects. The total binding free energy is defined as follows:

(1) $$\Delta G_{bind}=\Delta H-T\Delta S$$

### 3.6. M/GBSA

The enthalpy (ΔH) is composed of gas-phase energy ($\Delta G_{gas}$) and solvation energy ($\Delta G_{sol}$):

(2) $$\Delta G_{bind}=\Delta G_{gas}+\Delta G_{sol}$$

Gas phase energy ($\Delta G_{gas}$) includes electrostatic interactions ($\Delta E_{ele}$) and van der Waals energy ($\Delta E_{vdW}$), while the solvation energy ($\Delta G_{sol}$) is split into a polar term ($\Delta G_{gb}$) and a nonpolar term ($\Delta G_{np}$).

(3) $$\Delta G_{gas}=\Delta E_{ele}+\Delta E_{vdW}$$

(4) $$\Delta G_{sol}=\Delta G_{gb}+\Delta G_{np}$$

The polar contribution ($\Delta G_{gb}$) is obtained by solving the Generalized Born equation, while the non-polar term ($\Delta G_{np}$) is estimated using the solvent accessible surface area (SASA) [62] model:

(5) $$\Delta G_{np}=\gamma \Delta SASA+\beta$$

For MM/GBSA calculations, the parameter used are as follows [63]:

$$\begin{array}{c} \gamma =0.0072\;\mathrm{kcal}/\mathrm{mol}\cdot {Å}^{2}\\ \beta =0\;\mathrm{kcal}/\mathrm{mol} \end{array}$$

### 3.7. The Interaction Entropy (IE) Method

The entropy contribution is determined via the IE method [38]:

(6) $$-T\Delta S=KTln\left\langle e^{\beta \Delta E_{pl}^{int}}\right\rangle$$

where $E_{pl}^{int}$ represents the protein–ligand interaction energy, and $\beta =\frac{1}{KT}$, the fluctuation in interaction energy relative to the mean interaction energy ($\Delta E_{pl}^{int}$), is given by the following equation:

(7) $$\Delta E_{pl}^{int}=E_{pl}^{int}-\left\langle E_{pl}^{int}\right\rangle$$

### 3.8. The Alanine Scanning Interaction Entropy (ASIE) Method

The contribution of a specific residue (x) to binding energy is assessed using the alanine scanning approach [64], where the residue is mutated to alanine (a):

(8) $$\begin{array}{c} \Delta \Delta G_{bind}^{x\to a}=\Delta G_{bind}^{x}-\Delta G_{bind}^{a}=\Delta \Delta H^{x-a}-T\Delta \Delta S^{x-a} \end{array}$$

(9) $$\Delta \Delta H^{x-a}-T\Delta \Delta S^{x-a}=\left(\Delta H^{x}-T\Delta S^{x}\right)-\left(\Delta H^{a}-T\Delta S^{a}\right)$$

Here, the enthalpy term (ΔH) is obtained from MM/GBSA, while the entropic component (−TΔS) is computed using the IE method.

## 4. Conclusions

This study demonstrates a comprehensive computational approach to identify and analyze potential small-molecule inhibitors targeting PCSK9, a critical regulator of LDL-c levels implicated in atherosclerosis and cardiovascular diseases. Through a combination of high-throughput virtual screening, molecular docking, molecular dynamics simulations, and binding free energy calculations, we identified a novel inhibitor (AK-968/12164422) exhibiting comparatively strong binding affinity and stability within the PCSK9 binding pocket. Combined with two known active compounds (SBC and RIm), binding free energy and interaction mechanisms with the PCSK9 protein were investigated. Due to the absence of well-defined pockets in PCSK9, the polarization effect, which more accurately describes the non-bonded interaction between the protein and inhibitor, is found to be critically important and was included in the MD simulations. For the RIm molecule, hydrophobic interactions or π–π stacking were mainly formed and maintain the stability of the PCSK9–RIm structure. It is different from the SBC and AK molecules, in which significant hydrogen bonding and hydrophobic interactions are both critical factors maintaining the stability of the protein–ligand complex structure. To predict the strength of the inhibitor’s tendency to interact with PCSK9, the binding free energy of PCSK9 with each inhibitor was calculated. The binding free energy between PCSK9 and the small molecule SBC is the strongest, with the energy of −11.28 kcal/mol; the binding affinities of the RIm and AK systems are close to each other with values of −3.39 and −5.77 kcal/mol, respectively. For comparison, the reported Kd value for a PCSK9 inhibitor, the peptide-based inhibitor Pep2-8, is 0.7 µM with a ΔG ≈ −8.5 kcal/mol. The Kd of a novel small-molecule inhibitor, compound 13, reported by Sun et al. [26], is 2.50 μM with a ΔG of −7.6 kcal/mol. The not very high binding affinity indicates the significant challenges of small-molecule drug design targeting the PCSK9–LDLR PPI interface, which lacks well-defined pockets or grooves. The identified small molecule inhibitor AK may serve as a promising lead compound for further optimization. The contribution of each residue to the binding free energy was calculated to understand the source of the difference in the binding affinity among three inhibitors. Residue PHE379 was detected as the hot-spot residue among the three systems, which is consistent with the π–π stacking formed in three systems. In addition, residues ARG237 and GLU195 are the common hot-spot residues for the protein bound to the SBC and AK molecules. It is consistent with the ASIE calculation, in which the energy contribution of each individual residue was calculated. It is worthy to note that the total energy contribution of residues within 5 Å of the binding interface in PCSK9–RIm system was −4.16 kcal/mol, which is significantly distinctly lower than the energy contribution of the residues in the other two systems (PCSK9–SBC: −9.15 kcal/mol, PCSK9–AK: −13.47 kcal/mol), and PCSK9–AK exhibited the strongest binding affinity. It revealed that the hot-spot residues in the protein, which either directly or indirectly participate in the binding, significantly impact and determine the binding affinity between the protein and inhibitor. Although the compound is a potential inhibitor, as no in vitro assays were performed in this study to confirm its biological activity, this study is expected to contribute to the rational design of small-molecule therapeutics for managing hypercholesterolemia and preventing cardiovascular diseases.

## Figures and Tables

**Table 1:** Pharmacokinetic and physiological properties of the three ligands.

Molecule	MW (g/mol)	AMES Toxicity	Carcinogenicity	Acute Oral Toxicity	Rat Acute Toxicity (LD50, mol/kg)	Rotatable Bonds	H-Bond Acceptors	H-Bond Donors	ESOL Log S	TPSA	W Log P	Log Kp (cm/s)
SBC	527.61	None	None	III	2.70	10	8	1	−5.17	92.2	3.03	−6.84
RIm	495.62	None	None	III	2.76	11	5	1	−4.64	74.72	4.87	−7.16
AK	447.52	None	None	III	2.23	7	8	3	−5.29	121.46	3.31	−6.56

**Table 2:** The binding free energy and each energy term in the three PCSK9–inhibitor complexes. Errors are expressed as the standard error of the mean, and all values are in kcal/mol.

System	Force Field Replica		ΔE_vdw_	ΔE_ele_	ΔG_gb_	ΔG_np_	ΔH	−TΔS	ΔG_bind_
SBC	AMBER	No. 1	−23.21 ± 0.16	−20.72 ± 0.25	26.85 ± 0.16	−2.36 ± 0.01	−19.43 ± 0.11	15.97 ± 0.55	−3.46 ± 0.33
		No. 2	−23.24 ± 0.16	−19.95 ± 0.24	26.09 ± 0.13	−2.29 ± 0.01	−19.38 ± 0.11	16.09 ± 0.38	−3.29 ± 0.24
		No. 3	−28.08 ± 0.15	−20.77 ± 0.59	30.66 ± 0.42	−2.70 ± 0.01	−20.89 ± 0.17	25.95 ± 0.56	5.06 ± 0.37
		Average	−24.84 ± 0.16	−20.48 ± 0.36	27.87 ± 0.24	−2.45 ± 0.01	−19.90 ± 0.13	19.34 ± 0.50	−0.56 ± 0.31
	PPC	No. 1	−36.22 ± 0.20	−27.99 ± 0.17	40.06 ± 0.14	−3.29 ± 0.02	−27.44 ± 0.14	8.63 ± 0.29	−18.81 ± 0.21
		No. 2	−20.51 ± 0.14	−23.45 ± 0.16	27.09 ± 0.12	−2.25 ± 0.01	−19.11 ± 0.10	14.57 ± 0.59	−4.54 ± 0.34
		No. 3	−22.60 ± 0.15	−35.96 ± 0.20	38.14 ± 0.14	−2.48 ± 0.01	−22.89 ± 0.12	12.39 ± 0.57	−10.50 ± 0.34
		Average	−26.44 ± 0.16	−29.13 ± 0.18	35.10 ± 0.13	−2.67 ± 0.01	−23.15 ± 0.12	11.86 ± 0.48	−11.28 ± 0.30
RIm	AMBER	No. 1	−28.83 ± 0.17	−26.81 ± 0.49	59.51 ± 0.56	−2.37 ± 0.01	1.50 ± 0.15	42.55 ± 0.59	44.05 ± 0.37
		No. 2	−26.08 ± 0.18	−15.52 ± 0.59	43.28 ± 0.67	−2.08 ± 0.01	−0.40 ± 0.16	50.48 ± 0.54	50.08 ± 0.35
		No. 3	−29.28 ± 0.23	−16.61 ± 0.56	50.96 ± 0.53	−2.46 ± 0.02	2.62 ± 0.26	48.65 ± 0.48	51.27 ± 0.37
		Average	−28.06 ± 0.19	−19.64 ± 0.54	51.25 ± 0.59	−2.30 ± 0.01	1.24 ± 0.19	47.23 ± 0.54	48.47 ± 0.36
	PPC	No. 1	−26.64 ± 0.15	−11.02 ± 0.14	24.11 ± 0.16	−2.59 ± 0.02	−16.13 ± 0.11	10.51 ± 0.57	−5.62 ± 0.34
		No. 2	−34.39 ± 0.17	−9.08 ± 0.21	29.81 ± 0.22	−3.16 ± 0.01	−16.83 ± 0.12	13.48 ± 0.51	−3.35 ± 0.32
		No. 3	−33.26 ± 0.25	−13.86 ± 0.19	27.01 ± 0.20	−2.95 ± 0.02	−23.07 ± 0.23	21.85 ± 0.50	−1.22 ± 0.36
		Average	−31.43 ± 0.19	−11.32 ± 0.18	26.98 ± 0.19	−2.90 ± 0.02	−18.67 ± 0.15	15.28 ± 0.53	−3.39 ± 0.34
AK	AMBER	No. 1	−26.00 ± 0.12	−28.67 ± 0.38	42.42 ± 0.34	−2.39 ± 0.01	−14.65 ± 0.11	24.79 ± 0.48	10.14 ± 0.30
		No. 2	−35.80 ± 0.15	−33.01 ± 0.43	48.15 ± 0.40	−3.31 ± 0.01	−23.96 ± 0.17	28.41 ± 0.59	4.45 ± 0.38
		No. 3	−24.58 ± 0.17	−55.48 ± 0.56	52.01 ± 0.48	−2.43 ± 0.01	−30.48 ± 0.21	37.82 ± 0.59	7.34 ± 0.40
		Average	−28.80 ± 0.15	−39.05 ± 0.46	47.53 ± 0.41	−2.71 ± 0.01	−23.03 ± 0.16	30.34 ± 0.55	7.31 ± 0.36
	PPC	No. 1	−26.72 ± 0.15	−42.13 ± 0.48	45.72 ± 0.43	−2.29 ± 0.01	−25.43 ± 0.15	23.43 ± 0.42	−1.99 ± 0.29
		No. 2	−26.69 ± 0.17	−56.91 ± 0.37	58.96 ± 0.31	−2.79 ± 0.01	−27.43 ± 0.17	23.71 ± 0.35	−3.72 ± 0.26
		No. 3	−33.89 ± 0.16	−40.74 ± 0.35	49.17 ± 0.31	−3.23 ± 0.01	−28.69 ± 0.15	17.08 ± 0.42	−11.61 ± 0.29
		Average	−29.10 ± 0.16	−46.59 ± 0.40	51.28 ± 0.35	−2.77 ± 0.01	−27.18 ± 0.16	21.41 ± 0.40	−5.77 ± 0.28

## Data Availability

Data are contained within the article.

## Supplementary Materials

The following supporting information can be downloaded at: https://www.mdpi.com/article/10.3390/molecules30142962/s1, Figure S1: The variation of root mean squared deviation (RMSD) along with simulation time for three complex systems under PPC force fields; Figure S2: (A) Hydrogen bond proportion in three replicas between SBC-115076 and PCSK9. (B) Interactions between ligand and residues of PCSK9 in binding sites; Figure S3. (A) Hydrogen bond proportion in three replicas between RIm13 and PCSK9. (B) Interactions between ligand and residues of PCSK9 in binding sites; Figure S4. Superposition of representative structures from the second populated conformational cluster for the SBC (green), RIm (orange), and AK (wheat) systems; Figure S5. Superposition of the initial docking pose (green sticks) and two representative structures from the most populated cluster 1 (orange sticks), cluster 2 (yellow sticks) for ligands (A) SBC, (B) RIm, and (C) AK. The protein receptor is shown as a semi-transparent grey surface; Figure S6. The DCCM was calculated by the coordinates of Cα atoms for the protein binding with SBC, RIm and AK under replica (A) 2 and (B) 3. (C) Correlation of PCSK9 residues in three systems. Top 5 residues with the highest correlation were labeled; Table S1: The average energy terms of binding free energy for hotspot mutated residues of PCSK9 in three systems calculated by ASIE method. All values are in kcal/mol.
