# Supplementary material for: Insights into Binding Mechanisms of Potential Inhibitors Targeting PCSK9 Protein via Molecular Dynamics Simulation and Free Energy Calculation
Source: Molecules. 2025 Jul 14;30(14):2962. doi: 10.3390/molecules30142962 (PMC12298945; doi:10.3390/molecules30142962)
Supplement: Supplementary file 1 [file molecules-30-02962-s001.zip › molecules-3717751-supplementary.pdf]

Insights into Binding Mechanisms of Potential Inhibitors Targeting PCSK9 protein via Molecular dynamics simulation and free energy calculation

Xingyu Wu[1], Xi Zhu[1], Min Fang[1], Fenghua Qi[2], Zhixiang Yin[1], John Z. H. Zhang[3,4,5], Shihua Luo[6*], Tong Zhu[5,7*], and Ya Gao[1*]

[1]School of Mathematics, Physics and Statistics, Shanghai University of Engineering Science, Shanghai 201620, China

[2] School of Electronic Engineering, Nanjing Xiaozhuang University, Nanjing 211171, China

[3] Faculty of Synthetic Biology, Shenzhen University of Advanced Technology, Shenzhen 518107, China

[4] Key Laboratory of Quantitative Synthetic Biology, Shenzhen Institute of Synthetic Biology, Shenzhen Institutes of Advanced Technology, Chinese Academy of Sciences, Shenzhen 518055, China

[5]NYU-ECNU Center for Computational Chemistry at NYU Shanghai, Shanghai 200062, China

[6]Department of Traumatology, Rui Jin Hospital, School of Medicine, Shanghai Jiao Tong University, Shanghai 200025, China

[7]Shanghai Engineering Research Center of Molecular Therapeutics & New Drug Development, School of Chemistry and Molecular Engineering, East China Normal University, Shanghai 200062, China

*Corresponding author e-mail:

jqab@163.com; tzhu@lps.ecnu.edu.cn; gaoya@sues.edu.cn

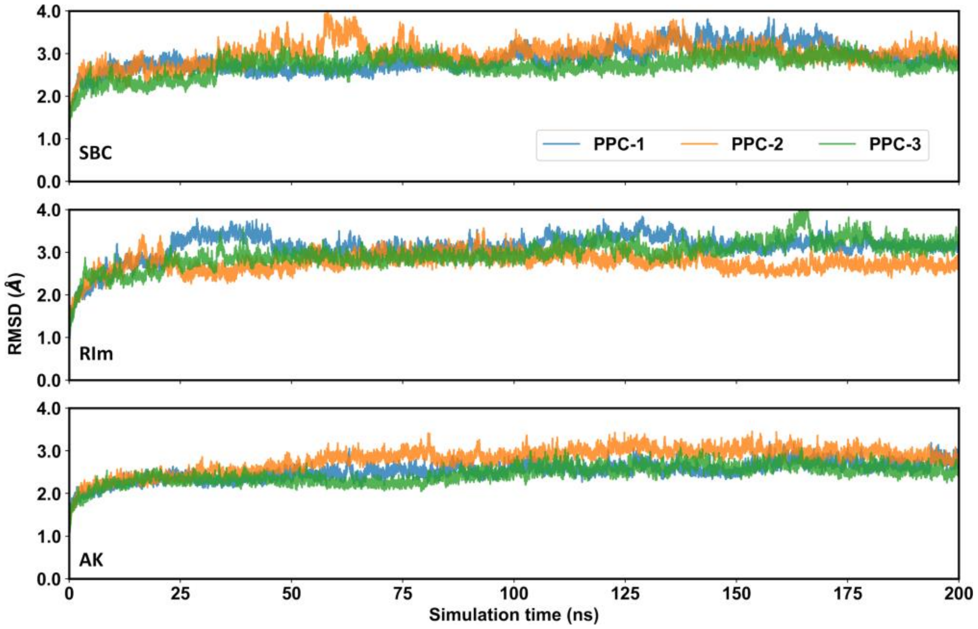

Figure S1. The variation of root mean squared deviation (RMSD) along with simulation time for three complex systems under PPC force fields.

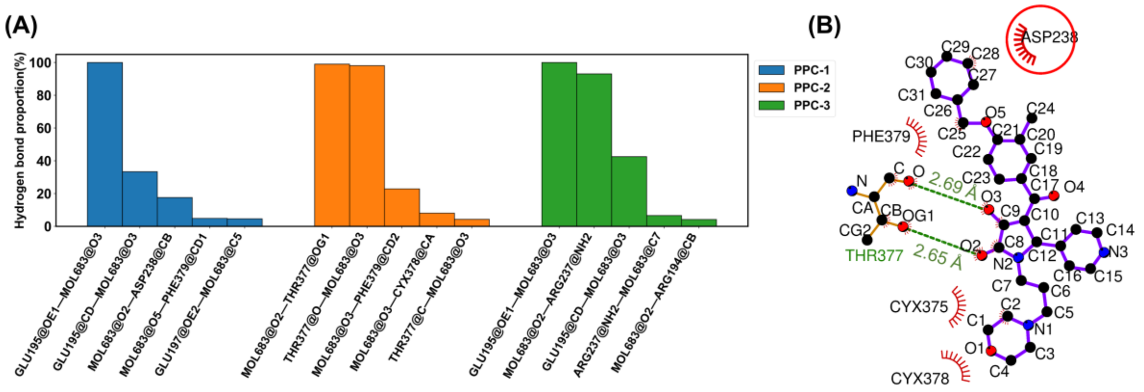

Figure S2. (A) Hydrogen bond proportion in three replicas between SBC-115076 and PCSK9**. (B)** Interactions between ligand and residues of PCSK9 in binding sites.

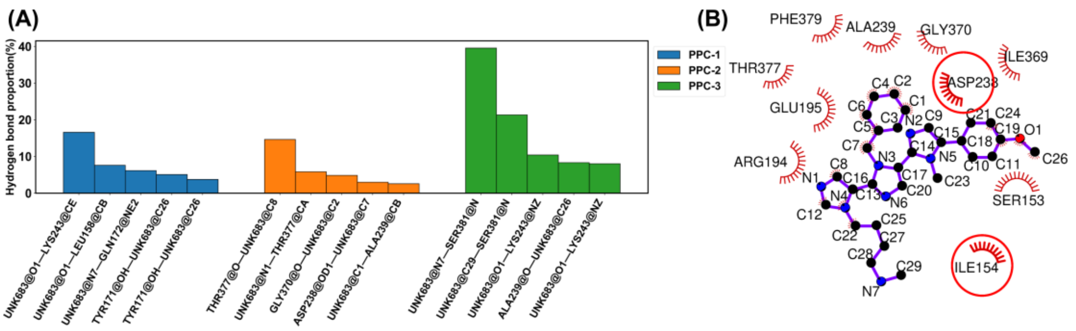

Figure S3. (A) Hydrogen bond proportion in three replicas between RIm13 and PCSK9**. (B)** Interactions between ligand and residues of PCSK9 in binding sites.

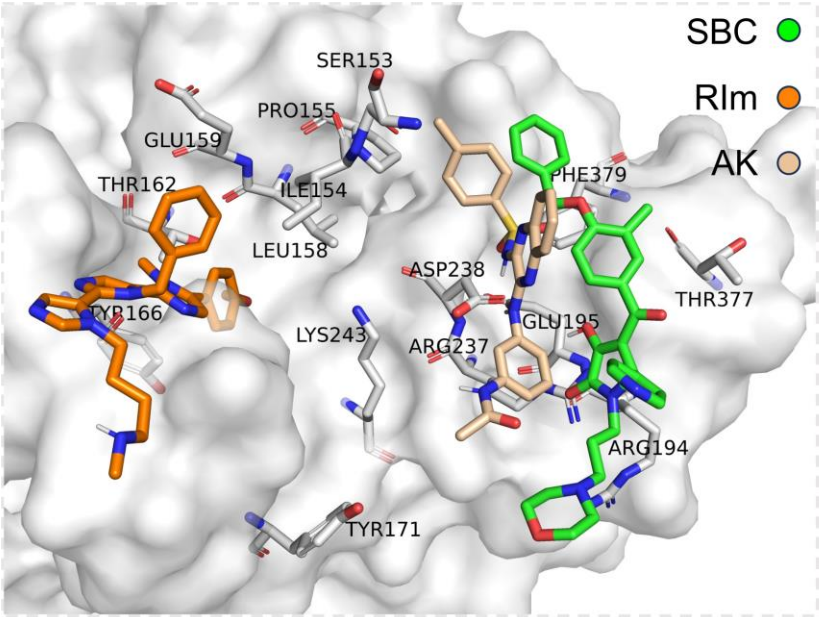

Figure S4. Superposition of representative structures from the second populated conformational cluster for the SBC (green), RIm (orange), and AK (wheat) systems.

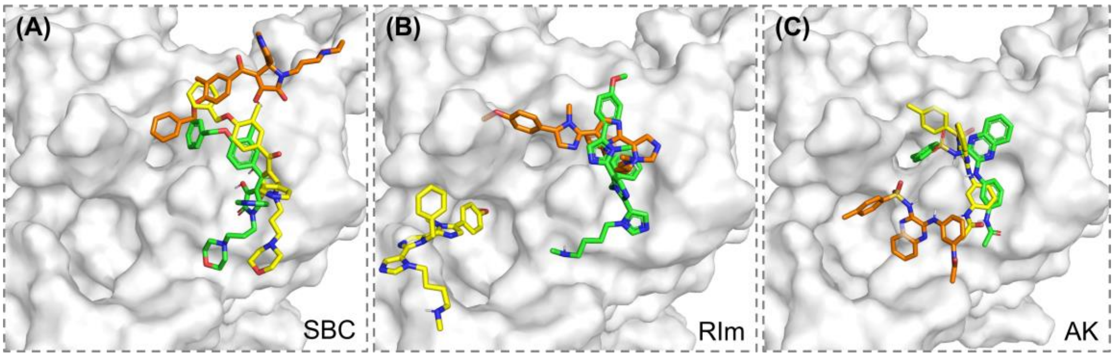

Figure S5. Superposition of the initial docking pose (green sticks) and two representative structures from the most populated cluster 1 (orange sticks), cluster 2 (yellow sticks) for ligands **(A)** SBC, **(B)** RIm, and **(C)** AK. The protein receptor is shown as a semi-transparent grey surface.

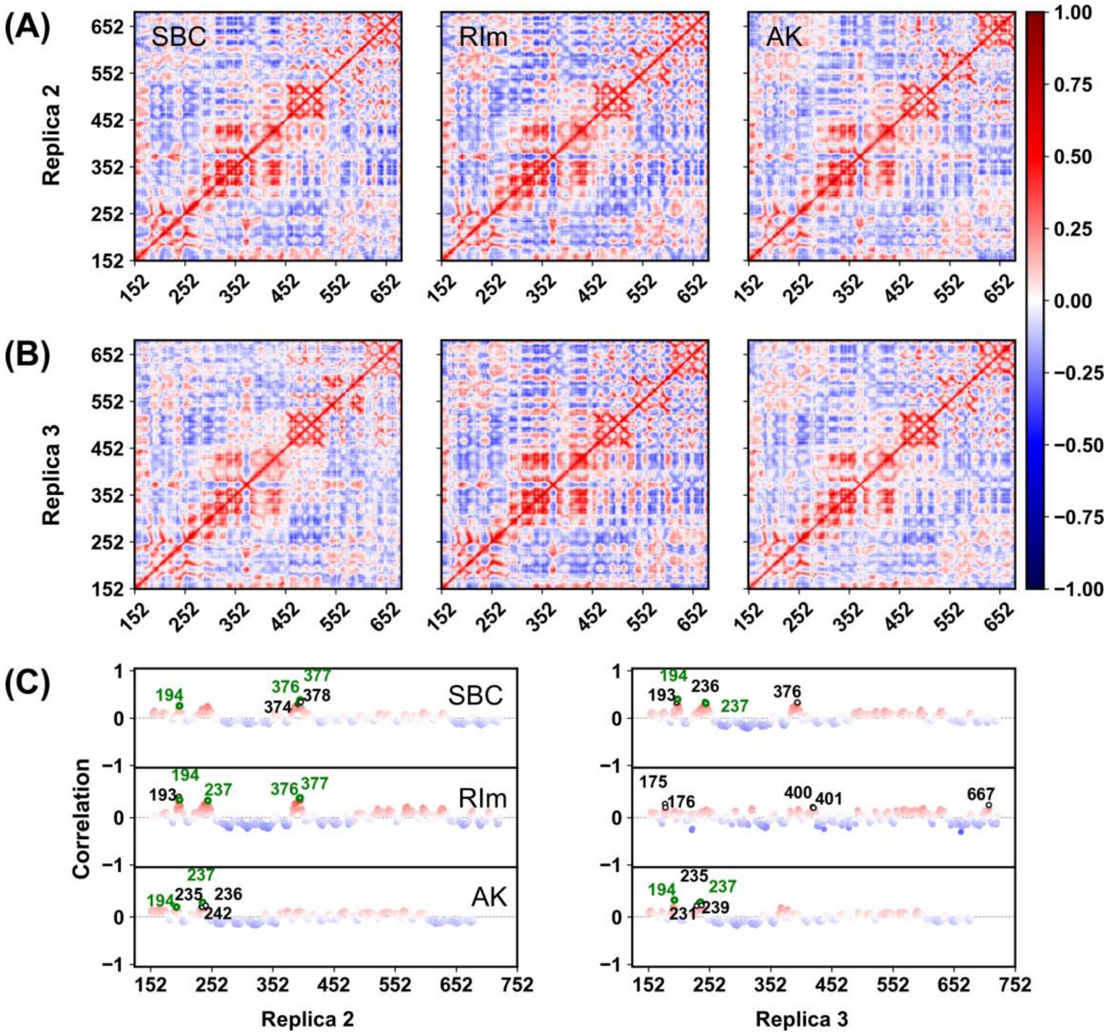

Figure S6. The DCCM was calculated by the coordinates of Cα atoms for the protein binding with SBC, RIm and AK under replica **(A)** 2 and **(B)** 3. (**C**) Correlation of PCSK9 residues in three systems. Top 5 residues with the highest correlation were labeled.

Table S1. The average energy terms of binding free energy for hotspot mutated residues of PCSK9 in three systems calculated by ASIE method. All values are in kcal/mol.

Systems	Simulation	Residues	$\Delta\Delta E_{vdw}$	$\Delta\Delta E_{ele}$	$\Delta\Delta G_{gb}$	$\Delta\Delta G_{np}$	$\Delta\Delta H$	$-T\Delta\Delta S$	$\Delta\Delta G_{bind}$
SBC	AMBER	PRO155	-0.87 ± 0.69	-0.02 ± 0.04	0.12 ± 0.09	-0.09 ± 0.03	-0.85 ± 0.66	0.82 ± 0.62	-0.03 ± 0.96
		PRO173	0.00 ± 0.00	0.00 ± 0.01	0.00 ± 0.00	0.00 ± 0.00	0.00 ± 0.00	0.00 ± 0.00	0.00 ± 0.00
		HID193	-0.07 ± 0.04	-0.12 ± 0.12	0.14 ± 0.13	0.00 ± 0.00	-0.05 ± 0.05	0.00 ± 0.00	-0.05 ± 0.05
		ARG194	-0.07 ± 0.04	0.62 ± 0.28	-0.52 ± 0.27	0.00 ± 0.00	0.03 ± 0.03	0.01 ± 0.02	0.04 ± 0.01
		GLU195	0.07 ± 0.42	-5.93 ± 5.88	4.18 ± 2.62	-0.01 ± 0.01	-1.68 ± 2.86	1.30 ± 2.23	-0.38 ± 0.63
		ARG237	-0.45 ± 0.71	-0.21 ± 2.76	-0.02 ± 2.28	-0.05 ± 0.09	-0.73 ± 1.27	0.41 ± 0.72	-0.32 ± 0.56
		ASP238	-0.49 ± 0.33	-2.96 ± 0.38	3.65 ± 0.82	-0.05 ± 0.05	0.16 ± 0.10	0.85 ± 1.33	1.00 ± 1.43
		LYS243	-0.07 ± 0.06	1.38 ± 0.01	-1.28 ± 0.06	-0.00 ± 0.01	0.04 ± 0.01	0.01 ± 0.01	0.04 ± 0.02
		ILE369	-1.78 ± 0.54	-0.07 ± 0.03	0.39 ± 0.10	-0.20 ± 0.08	-1.66 ± 0.55	0.94 ± 0.29	-0.73 ± 0.46
		THR377	0.47 ± 0.84	-3.73 ± 3.18	1.51 ± 1.01	-0.05 ± 0.01	-1.79 ± 1.34	1.33 ± 1.22	-0.46 ± 0.87
		CYX378	-0.78 ± 0.57	-0.06 ± 0.08	0.32 ± 0.24	-0.03 ± 0.01	-0.56 ± 0.42	0.37 ± 0.33	-0.18 ± 0.24
		PHE379	-3.56 ± 0.44	-0.45 ± 0.29	1.19 ± 0.09	-0.21 ± 0.07	-3.04 ± 0.19	1.10 ± 0.51	-1.94 ± 0.35
		**TOTAL**	**-7.61 ± 1.58**	**-11.55 ± 5.59**	**9.69 ± 4.75**	**-0.67 ± 0.15**	**-10.14 ± 2.57**	**7.14 ± 3.46**	**-3.00 ± 1.07**
	PPC	ASP175	-0.06 ± 0.10	-0.77 ± 0.68	0.77 ± 0.67	-0.01 ± 0.01	-0.07 ± 0.12	0.02 ± 0.04	-0.04 ± 0.08
		HID193	-0.43 ± 0.38	-0.39 ± 0.64	0.45 ± 0.67	-0.01 ± 0.01	-0.37 ± 0.34	0.10 ± 0.16	-0.27 ± 0.18
		ARG194	-2.20 ± 1.86	2.53 ± 1.71	-1.61 ± 0.99	-0.15 ± 0.13	-1.42 ± 1.26	0.35 ± 0.45	-1.07 ± 0.99
		GLU195	1.73 ± 1.71	-16.73 ± 12.73	10.17 ± 7.02	-0.09 ± 0.10	-4.91 ± 4.73	3.69 ± 3.24	-1.22 ± 3.00
		GLU197	-0.56 ± 0.54	-1.92 ± 3.03	1.86 ± 3.76	-0.02 ± 0.04	-0.64 ± 0.75	0.21 ± 0.32	-0.43 ± 0.85
		ARG237	-1.97 ± 1.71	-2.56 ± 7.71	1.25 ± 5.30	-0.17 ± 0.17	-3.46 ± 3.97	0.88 ± 1.50	-2.58 ± 2.58

		ASP238	-0.59 ± 0.38	-2.99 ± 3.06	4.05 ± 2.28	-0.05 ± 0.04	0.42 ± 0.52	0.03 ± 0.08	0.45 ± 0.44
		ILE369	-1.08 ± 0.55	-0.01 ± 0.03	0.24 ± 0.10	-0.15 ± 0.08	-1.00 ± 0.56	0.50 ± 0.19	-0.50 ± 0.37
		SER372	-0.01 ± 0.01	0.01 ± 0.04	0.00 ± 0.00	0.00 ± 0.00	-0.00 ± 0.03	0.00 ± 0.00	-0.00 ± 0.03
		THR377	-0.60 ± 1.41	-2.32 ± 3.23	1.09 ± 0.95	-0.05 ± 0.04	-1.86 ± 1.01	0.25 ± 0.06	-1.62 ± 1.04
		CYX378	-0.39 ± 0.60	0.02 ± 0.03	0.11 ± 0.26	-0.01 ± 0.02	-0.28 ± 0.37	0.12 ± 0.20	-0.16 ± 0.17
		PHE379	-3.16 ± 1.20	-0.57 ± 0.19	1.35 ± 0.13	-0.20 ± 0.10	-2.59 ± 1.06	0.89 ± 0.32	-1.70 ± 0.76
		**TOTAL**	**-9.30 ± 2.60**	**-25.72 ± 13.49**	**19.73 ± 9.50**	**-0.90 ± 0.40**	**-16.19 ± 6.98**	**7.04 ± 4.32**	**-9.15 ± 2.65**
**Rlm**	**AMBER**	SER153	-0.08 ± 0.00	-0.01 ± 0.00	0.03 ± 0.00	-0.01 ± 0.00	-0.06 ± 0.00	-0.04 ± 0.00	-0.11 ± 0.00
		ILE154	-0.62 ± 0.00	0.11 ± 0.00	0.24 ± 0.00	-0.05 ± 0.00	-0.30 ± 0.00	0.42 ± 0.00	0.12 ± 0.00
		PRO155	-1.79 ± 0.00	-0.07 ± 0.00	1.04 ± 0.00	-0.07 ± 0.00	-0.89 ± 0.00	1.04 ± 0.00	0.14 ± 0.00
		LEU158	-0.42 ± 0.00	0.06 ± 0.00	0.20 ± 0.00	-0.02 ± 0.00	-0.17 ± 0.00	0.06 ± 0.00	-0.10 ± 0.00
		HID193	-0.07 ± 0.05	-0.07 ± 0.09	0.12 ± 0.12	0.00 ± 0.00	-0.03 ± 0.03	0.01 ± 0.01	-0.02 ± 0.03
		ARG194	-0.17 ± 0.11	0.04 ± 0.28	-0.00 ± 0.23	-0.01 ± 0.01	-0.15 ± 0.07	0.13 ± 0.03	-0.02 ± 0.08
		GLU195	-0.18 ± 0.03	-0.37 ± 0.27	0.34 ± 0.23	-0.00 ± 0.01	-0.22 ± 0.02	0.05 ± 0.03	-0.16 ± 0.04
		ARG237	-0.04 ± 0.01	0.17 ± 0.21	-0.16 ± 0.16	-0.00 ± 0.01	0.00 ± 0.01	0.01 ± 0.01	0.01 ± 0.01
		ASP238	-0.40 ± 0.10	-0.76 ± 0.25	0.72 ± 0.22	-0.04 ± 0.02	-0.48 ± 0.08	0.07 ± 0.40	-0.41 ± 0.41
		LYS243	-0.04 ± 0.04	0.38 ± 0.19	-0.32 ± 0.16	0.00 ± 0.00	0.02 ± 0.02	0.02 ± 0.03	0.04 ± 0.02
		ILE369	-3.31 ± 0.71	-0.07 ± 0.10	2.41 ± 1.08	-0.27 ± 0.04	-1.24 ± 0.54	9.36 ± 13.77	8.12 ± 14.12
		SER376	-0.01 ± 0.01	0.04 ± 0.02	-0.02 ± 0.03	0.00 ± 0.00	0.00 ± 0.00	0.00 ± 0.01	0.00 ± 0.01
		THR377	-0.17 ± 0.17	-0.02 ± 0.13	0.12 ± 0.14	-0.01 ± 0.02	-0.08 ± 0.14	0.25 ± 0.22	0.18 ± 0.14
		PHE379	-3.25 ± 1.04	-0.70 ± 0.61	3.17 ± 1.22	-0.17 ± 0.04	-0.96 ± 0.37	1.48 ± 1.29	0.53 ± 1.00

		**TOTAL**	**-9.28 ± 1.24**	**1.45 ± 5.49**	**4.51 ± 4.54**	**-1.62 ± 1.78**	**-2.66 ± 2.31**	**12.22 ± 12.56**	**9.55 ± 14.87**
		ASP175	-0.01 ± 0.01	-0.05 ± 0.02	0.05 ± 0.02	0.00 ± 0.00	-0.01 ± 0.00	0.00 ± 0.00	-0.01 ± 0.00
		HID193	-0.09 ± 0.12	-0.03 ± 0.05	0.04 ± 0.06	0.00 ± 0.00	-0.08 ± 0.11	0.00 ± 0.00	-0.08 ± 0.11
		ARG194	-1.45 ± 2.49	-0.07 ± 0.08	0.11 ± 0.13	-0.11 ± 0.20	-1.53 ± 2.62	0.28 ± 0.48	-1.25 ± 2.14
		GLU195	-0.91 ± 1.39	-0.02 ± 0.07	0.07 ± 0.05	-0.03 ± 0.06	-0.89 ± 1.34	0.19 ± 0.31	-0.70 ± 1.03
		ARG237	-0.05 ± 0.05	0.04 ± 0.06	-0.04 ± 0.06	0.00 ± 0.00	-0.05 ± 0.04	0.00 ± 0.01	-0.04 ± 0.04
		ASP238	-0.65 ± 0.81	-0.24 ± 0.27	0.30 ± 0.35	-0.07 ± 0.10	-0.65 ± 0.83	0.24 ± 0.35	-0.41 ± 0.49
		LYS243	-0.76 ± 0.72	-0.08 ± 0.17	0.04 ± 0.12	-0.08 ± 0.09	-0.88 ± 0.85	0.38 ± 0.33	-0.49 ± 0.62
	**PPC**	ILE369	-2.02 ± 1.80	-0.06 ± 0.09	0.47 ± 0.44	-0.17 ± 0.15	-1.78 ± 1.57	1.01 ± 0.88	-0.77 ± 0.80
		SER372	-0.01 ± 0.01	-0.01 ± 0.04	0.01 ± 0.04	0.00 ± 0.00	-0.01 ± 0.01	0.00 ± 0.00	-0.01 ± 0.01
		CYX375	-0.02 ± 0.02	0.11 ± 0.10	-0.12 ± 0.11	0.00 ± 0.00	-0.03 ± 0.04	0.00 ± 0.00	-0.03 ± 0.04
		SER376	-0.01 ± 0.01	0.05 ± 0.05	-0.04 ± 0.04	0.00 ± 0.00	0.00 ± 0.00	0.00 ± 0.01	0.00 ± 0.00
		THR377	-0.14 ± 0.23	-0.13 ± 0.19	0.13 ± 0.18	-0.01 ± 0.01	-0.15 ± 0.25	0.02 ± 0.03	-0.13 ± 0.22
		CYX378	-0.02 ± 0.02	0.02 ± 0.03	-0.01 ± 0.01	0.00 ± 0.00	-0.01 ± 0.01	0.00 ± 0.02	-0.01 ± 0.02
		PHE379	-1.81 ± 1.56	-0.52 ± 0.54	1.07 ± 0.94	-0.12 ± 0.11	-1.38 ± 1.21	1.27 ± 1.14	-0.11 ± 0.54
		VAL380	-0.12 ± 0.18	0.03 ± 0.05	-0.08 ± 0.11	0.00 ± 0.00	-0.17 ± 0.24	0.04 ± 0.08	-0.13 ± 0.17
		**TOTAL**	**-8.05 ± 6.56**	**-0.95 ± 0.72**	**1.99 ± 1.62**	**-0.59 ± 0.47**	**-7.61 ± 6.21**	**3.44 ± 2.54**	**-4.16 ± 4.10**
		ILE154	-1.80 ± 1.91	0.11 ± 0.15	0.41 ± 0.39	-0.12 ± 0.12	-1.41 ± 1.51	0.24 ± 0.34	-1.17 ± 1.54
**AK**	**AMBER**	PRO155	-1.45 ± 1.31	0.15 ± 0.13	0.04 ± 0.06	-0.07 ± 0.06	-1.32 ± 1.18	0.68 ± 0.69	-0.65 ± 0.50
		ASN157	-0.25 ± 0.36	0.27 ± 0.29	-0.17 ± 0.17	-0.01 ± 0.02	-0.16 ± 0.27	0.00 ± 0.01	-0.16 ± 0.26
		LEU158	-1.04 ± 1.34	-0.07 ± 0.09	0.21 ± 0.27	-0.06 ± 0.09	-0.96 ± 1.25	0.03 ± 0.05	-0.93 ± 1.27

	PRO173	-0.29 ± 0.26	0.11 ± 0.17	-0.03 ± 0.07	-0.02 ± 0.02	-0.23 ± 0.18	0.02 ± 0.02	-0.21 ± 0.19
	ARG194	-0.90 ± 1.09	0.10 ± 1.38	0.34 ± 1.93	-0.07 ± 0.09	-0.54 ± 0.63	0.23 ± 0.38	-0.31 ± 0.29
	GLU195	-0.07 ± 0.90	-14.38 ± 20.47	7.94 ± 9.70	-0.04 ± 0.04	-6.55 ± 10.22	1.58 ± 2.58	-4.96 ± 7.64
	ARG237	-1.51 ± 1.67	-6.57 ± 13.01	5.59 ± 11.07	-0.12 ± 0.16	-2.61 ± 3.78	1.20 ± 1.84	-1.41 ± 1.95
	ASP238	-1.26 ± 0.52	-4.21 ± 7.10	4.80 ± 6.26	-0.12 ± 0.06	-0.79 ± 1.18	0.36 ± 1.08	-0.43 ± 0.30
	LYS243	-0.77 ± 0.57	-7.51 ± 14.77	6.11 ± 11.63	-0.10 ± 0.09	-2.26 ± 3.60	0.09 ± 0.12	-2.18 ± 3.67
	ILE369	-0.75 ± 0.35	0.14 ± 0.17	0.01 ± 0.06	-0.08 ± 0.04	-0.68 ± 0.30	0.68 ± 0.74	0.00 ± 0.67
	THR377	-0.35 ± 0.29	0.03 ± 0.34	0.12 ± 0.36	-0.03 ± 0.03	-0.23 ± 0.21	0.22 ± 0.34	-0.00 ± 0.33
	PHE379	-1.86 ± 0.77	-0.32 ± 0.27	0.88 ± 0.68	-0.21 ± 0.08	-1.51 ± 0.37	1.11 ± 0.37	-0.41 ± 0.63
	**TOTAL**	**-12.30 ± 4.87**	**-32.14 ± 25.56**	**26.25 ± 14.20**	**-1.05 ± 0.22**	**-19.24 ± 9.92**	**6.43 ± 3.12**	**-12.81 ± 7.22**
	ILE154	-0.67 ± 1.11	0.00 ± 0.03	0.16 ± 0.25	-0.08 ± 0.14	-0.59 ± 0.99	0.23 ± 0.39	-0.37 ± 0.60
	PRO155	-0.25 ± 0.20	0.02 ± 0.06	0.02 ± 0.05	-0.02 ± 0.03	-0.24 ± 0.21	0.06 ± 0.07	-0.18 ± 0.19
	ASN157	-0.03 ± 0.04	-0.10 ± 0.29	0.11 ± 0.32	0.00 ± 0.00	-0.01 ± 0.02	0.00 ± 0.00	-0.01 ± 0.02
	LEU158	-0.17 ± 0.21	-0.01 ± 0.05	-0.04 ± 0.03	-0.01 ± 0.02	-0.23 ± 0.31	0.03 ± 0.05	-0.20 ± 0.26
	PRO173	-0.03 ± 0.05	-0.02 ± 0.01	0.02 ± 0.03	0.00 ± 0.00	-0.03 ± 0.04	0.01 ± 0.02	-0.02 ± 0.03
**PPC**	ARG194	-1.02 ± 0.79	-2.65 ± 3.13	3.06 ± 3.15	-0.09 ± 0.07	-0.70 ± 0.60	0.65 ± 0.68	-0.05 ± 0.31
	GLU195	0.61 ± 0.69	-19.92 ± 18.93	8.58 ± 9.61	-0.01 ± 0.06	-10.74 ± 8.67	3.08 ± 2.64	-7.66 ± 6.04
	ARG237	-2.56 ± 1.31	-4.51 ± 2.87	4.82 ± 2.82	-0.17 ± 0.12	-2.43 ± 1.05	1.43 ± 0.49	-1.00 ± 0.58
	ASP238	-0.27 ± 2.74	-12.45 ± 16.63	8.77 ± 6.39	-0.12 ± 0.04	-4.08 ± 7.51	2.53 ± 3.42	-1.55 ± 4.10
	LYS243	-1.13 ± 1.62	-4.35 ± 12.34	2.85 ± 9.31	-0.06 ± 0.09	-2.69 ± 4.73	0.81 ± 1.33	-1.88 ± 3.40
	ILE369	-0.46 ± 0.69	0.01 ± 0.03	0.07 ± 0.06	-0.02 ± 0.03	-0.40 ± 0.63	0.23 ± 0.38	-0.17 ± 0.25

THR377	-0.56 ± 0.75	0.17 ± 0.31	-0.02 ± 0.16	-0.02 ± 0.02	-0.42 ± 0.60	0.17 ± 0.20	-0.25 ± 0.41
PHE379	-1.47 ± 1.23	-0.38 ± 0.44	1.01 ± 0.92	-0.14 ± 0.12	-0.97 ± 0.87	0.83 ± 0.82	-0.15 ± 0.06
**TOTAL**	**-8.01 ± 1.00**	**-44.19 ± 8.57**	**29.42 ± 4.50**	**-0.75 ± 0.10**	**-23.53 ± 3.29**	**10.06 ± 1.28**	**-13.47 ± 2.38**
